# Accuracy of Non-Contrast CT Brain Interpretation by Emergency Physicians: A cohort study

**DOI:** 10.12669/pjms.292.3220

**Published:** 2013-04

**Authors:** Anas Khan, Sami Qashqari, Abdul-Aziz Al-Ali

**Affiliations:** 1 Anas Khan, MBBS, SBEM, ABHS-EM, MHA, Emergency Medicine, Consultant, King Khalid University Hospital, King Saud University, Riyadh, Saudi Arabia.; 2 Sami Qashqari, MBBS, FRCP (C), Emergency Medicine, Consultant, King Khalid University Hospital, King Saud University, Riyadh, Saudi Arabia.; 3 Abdul-Aziz Al-Ali, MBBS, SBEM, ABHS-EM, Emergency Medicine, Chief Resident, King Faisal Specialist Hospital and Research Center, Riyadh, Saudi Arabia.

**Keywords:** Brain, Computed tomography (CT), Accuracy, Emergency, Interpretation

## Abstract

***Objective:*** To assess the accuracy of emergency physicians (EPs) in the interpretation of non-contrast CT Brain (NCCT Brain) by examining the inter rater reliability between EPs and radiology specialists.

***Methodology:*** A four months prospective cohort study was conducted at emergency department of King Khalid University Hospital (KKUH), Riyadh, Saudi Arabia. We studied the daily performance of our EPs, and compared it to the radiological report issued within the week after. Data were analyzed by calculating sensitivity, specificity, accuracy and agreement (kappa statistic), using radiology report as the reference standard.

***Results*** ***:*** Out of 241 cases eligible for the study, 210 (87.14%) were concordant, and 31 (12.86%) were discordant. The agreement (kappa) was to be 0.64.

***Conclusion*** ***:*** We concluded that our EPs are moderately accurate at interpreting NCCT Brain studies. Further education and training programs were necessary for all our EPs to improve the accuracy. Further studies are required to determine the most cost-effective method of minimizing consequential misinterpretations.

## INTRODUCTION

The use of Non-contrast CT (NCCT) Brain is a frequent radiological study and often becoming part of the screening tools in the emergency departments (EDs) for neurologic and traumatic complaints. It is required both in critical and non-critical cases. In traumas and other critical cases, time is the essence and the emergency physician (EP) must act quickly according to his ordered and related investigations. It is known fact that many EDs were functioning in a busy shift away from the formality of referrals and tracing report so as to minimize time and provide proper care to the patients. The use of NCCT in ED patients could be influential in clinical decision making by improving diagnostic confidence and also have impact on treatment plan. An EP with the capability of correctly interpreting NCCT could provide patient satisfaction, health service provider satisfaction and also managerial satisfaction.^1^

Review of studies about radiological images interpretation in EDs varies especially on different imaging modalities and the discrepancies between EPs and radiologists have been subject of study by different researchers. In a prospective study, at St George Hospital in Australia it was found that 190 out of 1282 scans were misinterpreted, with 78 had potential for acute consequences.^2^ An agreement of 86.6% was observed between EPs and radiologist from the review of 100 consecutive scans.^3^ In a study where 7 EPs and 14 registrars were examined 287 scans, in which it was found 32 were false negative.^4^ Differing findings were also present in relation to XRs and CTs.^5^^-^^7^ In a study of assessment of accuracy of EPs interpreting CXRs it was found that EPs frequently missed specific radiographic abnormalities, and there was considerable discrepancy between their interpretations when compared with trained radiologists.^8^ Not much published studies could be identified which shows an appropriate level of accuracy, in order to demonstrate a satisfactory competence in the interpretation of radiological images in general, or brain CTs in specific.

The objective of this study was to assess the accuracy of EPs at Emergency department of a referral hospital, in interpreting the NCCT Brain compared with radiology specialists.

## METHODOLOGY

This study was conducted at King Khalid University Hospital (KKUH), Riyadh. The hospital is located in the north-western of the Capital, serving a wide area around, with its ED receiving 160,000 visits annually. It’s an 800 beds tertiary training hospital and Trauma center continuously staffed by senior EPs, supervising training residents and interns. Realizing that the time is a critical issue, EPs immediately take the responsibility of interpreting the NCCT Brain they've ordered and take actions in the management of their patients. They do have a radiologist on call "out of duty hours", who could be in the hospital most of the time, but will usually responds to any in-hospital consultation even if he has to drive from home to the hospital, including any EPs inquiry about a radiological study. EPs at KKUH have been following this process for four years with no formal specialized radiological teaching sessions.

We conducted a prospective blinded cohort study of 255 adult patients presenting to KKUH-ED and undergoing plain NCCT brain as per EP discretion. Patients get NCCT Brain by an order from his treating EP who will need to inform the radiology technician on call. The nurse will fill the study form with: (*date, patient file number, the presenting complain, EP impression *and* disposition*). EPs interpreting the NCCT Brain are EM consultant, specialist or training resident. The forms are collected in a special box and after a week at least, the formal radiological report will be traced in the radiological electronic system. Reports are dictated by a certified radiology consultant or an assistant consultant. Inclusion criteria for the study include: any adult patient (>12 y. o. as per hospital regulations) undergoing plain NCCT Brain during the period started 1^st^ March till 30^th^ June 2009 at KKUH-ED. Exclusion criteria are NCCT Brain being interpreted by physicians other than EPs (radiology on call / admitting medical services). Then the EP interprets the NCCT Brain and writes his impression and disposition in the form provided by the nurse. The CT findings (Table-I) are important to be diagnosed on NCCT Brain, and considered missed finding if not written in the EP report form while reported by the radiology specialist. The forms are collected later from the designated box for the study. The radiology reports from the radiology system are traced. There was no specialized NCCT Brain interpretation teaching sessions conducted in the department prior the study.

Data were entered and analyzed by using the statistical software package SPSS (SPSS version 18.0, SPSS Inc., Chicago IL). By considering both a EPs report and radiologist report, inter rater reliability was assessed by calculating the sensitivity, specificity, accuracy and kappa coefficient, using the radiologist report as the reference standard. Kappa value of >0.75 considered as excellent agreement, 0.40-0.75 intermediate agreement, and <0.40 poor agreement. A p-value of <0.05 and 95% confidence intervals were used to indicate the statistical significance and prevision of the estimates.

**Table-I:** Classification of NCCT Brain diagnoses

Normal
Sub-arachnoid hemorrhage (SAH)
Intra-cerebral hemorrhage (ICH)
Sub-dural hemorrhage (SDH)
Epi-dural hemorrhage (EDH)
Ischemic stroke
Skull fracture
Space occupying lesion (SOL)
Brain edema
Mid-line shift
Hydrocephalus
Herniation
Brain contusion
Old stroke (infarction)
Pneumocephalus

**Table-II:** Distribution of Main symptom presentation

*Symptom*	*Frequency*	*%*
Head Trauma	78	32.1
Seizure	12	4.9
Loss of Consciousness (LOC)	14	5.8
Ataxia	2	0.8
Suspected Stroke	34	14.0
Headache	42	17.3
Suspected Meningitis	4	1.6
Vertigo / Dizziness	22	9.1
Confusion	13	5.3
Others	22	9.1
Total	243	100.0

**Table-III:** Agreement between EP and radiology reports

Concordance:	*210 cases* 185 normal cases 25 abnormal cases	*87.14 %*
Discordance:	*31 cases* 15 cases normal by the EP & abnormal by the radiologist 7 cases abnormal by the EP & normal by the radiologist 9 cases with mismatching abnormalities	*12.86 %*

**Table-IV:** Distribution of Discordance cases

15 cases: normal by the EP and abnormal by the radiologist	(6 cases: old strokes), (3 cases: brain edema), (4 cases: SOL), (1 case: hydrocephalus), (1 case: pneumocephaly).	
7 cases: abnormal by the EP and normal by the radiologist	(2 cases: hydrocephalus), (2 cases: old infarction), (1 case: SOL), (1 case: ICH), (1 case: brain edema).	
9 cases: with mismatching abnormalities between the EP and the radiologist	EP	Radiologist
	ICH	Contusion
	SAH, contusion	ICH, contusion
	SAH	SAH, SDH, Herniation
	SDH, pneumocephaly, Contusion	SDH, skull fracture
	Old infarction	ICH
	ICH, mid-line shift, SOL, brain edema	ICH, mid-line shift
	ICH, mid-line shift	ICH
	Skull fracture, SOL	Skull fracture
	Old infarction	Ischemic stroke

**Table-V:** Classification of EPs interpretation and Radiology report

*Radiology Report*			
EP interpretation	Normal	Abnormal	Total
Normal	185	15	200
Abnormal	7	25	32
Total	192	40	232

## RESULTS

During the four months period, 255 adult patients required NCCT Brain in the ED, as per EPs discretion. 12 cases were excluded because of insufficient data in the forms or in the radiology reports, making the total available reports 243.The frequency of main symptom that the patient presents with were given in Table-II. It shows that the most presenting symptom was trauma by 78 cases (32.1%), and the least was ataxia (0.8%).

From the 243 cases, EP solely interpreted 241 NCCT Brain, and took their decision in this regard. The two cases (0.8%) were excluded from the analysis, as they were referred for immediate radiology consultation. EP decided to discharge 175 patients (72.6%) on his own. The EP decided to refer 66 (27.4%) patients to other consulting services for further management.

The concordance analysis between EP and Radiologist shows that, they agreed upon 185 cases to be normal, and agreed on the abnormalities of another 25 cases. So, the agreement was observed in 210 cases (87.14 %). Disagreement (discordance) was noticed on 31 cases, whereas 15 cases reported normal by the EP were found to have abnormalities as per radiologist. Seven cases reported to have abnormalities by the EP but found to be normal by the radiologist. In nine cases incomplete or different abnormalities were reported among the EP and the radiologist. (Table-III)

Among the cases reported normal by the EP and found to be abnormal by the radiologist: six cases had old strokes in the radiology report. Half of them were referred for consulting services. Another three cases reported normal by the EP, and the radiology said there is brain edema. Two of them were discharged by the EP, and one was referred. EP reported four cases normal, while the radiologist found them to have SOL. EP discharged three of them and referred one. EP discharged one as normal, whereas later it was reported by the radiologist as hydrocephalus. A case reported normal by EP, but got referred, was reported by radiology to have pneumocephaly. The seven cases reported to have abnormalities by the EP and found to be normal by the radiologist were: two cases as hydrocephalus, both were discharged and the radiology report came back as normal. Two cases reported normal by the radiologist, reported old infarction by the EP. Another three cases were referred as abnormal (one each: brain edema, SOL and ICH), while the radiology report said they were normal. There were nine cases who got referred by the EPs because they interpreted abnormalities, which the radiologist found to have incomplete or different abnormalities (these are not included in Table-V) One case reported by the EP to be contusion, the radiology said it's ICH. Another case where the EP said it is SAH and contusion, the radiology report said it's ICH and contusion. A third case reported by the EP as SAH only, then the radiology report said there is SAH, SDH and herniation. In the fourth case, the radiology reported it as SDH and skull fracture, the EP interpreted as SDH, contusion and pneumocephaly. The fifth case had an ICH in the radiology report, and the EP said it is old infarction. The sixth case interpreted by the EP as ICH with a mid-line shift, while the radiologist added SOL and edema. The seventh case was interpreted by the EP to be ICH alone, where the radiologist added a mid-line shift. The eighth case the EP said it is skull fracture whereas the radiologist added a SOL. The last case, the EP found an ischemic stroke, while the radiologist reported it as an old one. (Table-IV)

The study results demonstrate that agreement between EPs and radiologist specialists’ interpretation of NCCT Brain scans is reasonable, with an overall concordance rate of 87.14%. For the analysis of 2 x 2 table (Table-V), the categories with the discordant positive findings (9 mismatching cases) were omitted. The observed agreement of NCCT brain by EPs as a diagnostic test compared with a reference standard of a radiologist report and other reliability measures obtained were:

Accuracy                                 :          90.5 (95% CI: 86.2--93.8)

Sensitivity                               :          96.3 (95% CI: 92.6--98.5)

Specificity                               :          62.5 (95% CI: 45.8--77.3)

Positive predictive value         :          92.5 (95% CI: 87.9 –- 95.7)

Negative predictive value        :          78.1 (95% CI: 60.0 -- 90.7)

Kappa                                      :          0.64 (95% CI: 0.50-- 0.78)

The kappa was (κ= 0.64), with p < 0.001, as it ranges between (0.5 – 0.8), which indicates that there is intermediate agreement between the EPs interpretation and radiology reports.

## DISCUSSION

The proper interpretation of abnormalities in NCCT brain by the EPs in the emergency department of any referral hospital will play an important role in providing appropriate timely care to the traumatic patients. This study has assessed the accuracy of EP in assessing the NCCT brain abnormalities when compared with the radiology reports. The results of this study demonstrate that EPs were able to interpret NCCT brain with a high degree of accuracy of 90.5% when compared with radiologists. A negative predictive value of 78.12% was calculated with the prevalence of abnormal NCCT brain scans in our study of 17.2% and a positive predictive value of 92.50% with the prevalence of normal NCCT brain scans was 82.76%. The false negative rate of our EPs was 3.6%.

Comparison with earlier published studies is not completely appropriate due to different use of methodologies and definitions of ‘normal’ and ‘abnormal’ scan. In 2003, Arendts *et al* at St George Hospital in Australia did a prospective similar study and found that 14.8% scans were misinterpreted, with 41.1% of these had potential for acute consequence.^2^ Mucci *et al* in Cumberland Hospital reviewed 100 consecutive scans and found agreement of 86.6% between the EPs and the Radiologists, with no findings that would change the overnight management.^3^ Khoo and Duffy examined 7 EPs and 14 registrars with a 287 scans, 32 were false negative.^4^

The overall accuracy of ED staff in the initial interpretation of radiographs has been studied earlier, in which it was reported the false negative rates ranges from 1% to 11%.^9^^-^^13^ Not having a consensus about the accepted limit of agreement for interpreting radiological studies in EDs is a major factor as it's not practically expected from the EPs to match the level of radiologists. And none of the studies has addressed the issue of inter observer agreement in the interpretation of NCCT brain scans between EPs and radiologist.

***Limitations of the study: ***Limitations of the study were: 1) Difficulties dealing with incomplete data in the forms (12 cases excluded) and the delay in radiological reporting. 2) EPs are aware of the clinical scenario of the case.

## CONCLUSION

Emergency Physicians at KKUH are moderately accurate in interpreting NCCT Brain in comparison to radiology specialists. Further studies are required to determine the most cost-effective method of minimizing consequential misinterpretations. NCCT Brain interpretations teaching sessions may further improve the EPs accuracy. Establishment of an appropriate level of accuracy is required as a benchmark.
